# Special Notice

**Published:** 1877-08-20

**Authors:** 


					﻿Editorial and Miscellaneous
SPECIAL NOTICE.
To all persons receiving this issue as a specimen,
we wish to say, try our journal. You will find it
plain and practical, containing a great many
valuable suggestions, hints, formulas, etc., which,
in the course of a year, will largely indemnify you
for the small amount of subscription. It is best
to take the back numbers, so as to have a com-
plete volume ; but may subscribe tor six months,
if you prefer. See our club and subscription rates
elsewhere.
				

